# Assessing the Functional Significance of Novel and Rare Variants of the *SLC26A4* Gene Found in Patients with Hearing Loss by Minigene Assay

**DOI:** 10.3390/ijms262110732

**Published:** 2025-11-04

**Authors:** Valeriia Yu. Danilchenko, Ekaterina A. Panina, Marina V. Zytzar, Konstantin E. Orishchenko, Olga L. Posukh

**Affiliations:** 1 Federal Research Center Institute of Cytology and Genetics, Siberian Branch of the Russian Academy of Sciences, Novosibirsk 630090, Russia; danilchenko_valeri@mail.ru (V.Y.D.); e.panina2@g.nsu.ru (E.A.P.); zytzar@bionet.nsc.ru (M.V.Z.); keor@bionet.nsc.ru (K.E.O.); 2 Novosibirsk State University, Novosibirsk 630090, Russia

**Keywords:** hearing loss, *SLC26A4*, splicing variants, minigene assay

## Abstract

The *SLC26A4* gene is one of the key genes involved in the etiology of hearing loss. It encodes pendrin, a transmembrane transporter protein functioning as a multifunctional anion exchanger. About 600 pathogenic *SLC26A4* variants are known to cause either nonsyndromic recessive hearing loss (DFNB4) or Pendred syndrome (hearing loss and thyroid dysfunction). While most pathogenic variants occur in coding regions and disrupt pendrin structure or function, about 25% are thought to impair splicing. For many, pathogenicity has been assessed only *in silico*, with limited experimental confirmation. We identified several novel and rare *SLC26A4* variants in patients with hearing loss from the Tyva and Altai Republics (Southern Siberia, Russia). Based on splicing predictions, six variants—intronic c.2034+1G>A, c.1545-168A>G, c.1708-125T>C, c.1708-18T>A, c.1804-31C>T, and exonic c.942A>G—were selected for analysis using a minigene assay. The results of *in vitro* analysis only partially matched *in silico* predictions: c.2034+1G>A caused aberrant splicing; c.1708-18T>A led to exon 16 skipping only in a small proportion of transcripts; the remaining variants showed no detectable splicing effect. These findings underscore the need for integrating *in silico* predictions with *in vitro* validation to accurately assess the functional impact of genetic variants, enabling their correct interpretation and reliable molecular diagnosis.

## 1. Introduction

Hereditary diseases are caused by various mutational changes in specific genes, and identifying such causative variants is essential for establishing an accurate molecular diagnosis. With the advent of modern genomic technologies, the accuracy and sensitivity of DNA diagnostics for detecting point mutations in coding regions have improved considerably. However, the contribution of variants affecting splicing to the etiology of hereditary disorders has long been underestimated [1,2,3,4].

Splicing is a complex process carried out by the spliceosome—a ribonucleoprotein complex that recognizes multiple splicing signals. Variants in either introns or exons can disrupt correct mRNA processing by altering canonical splice sites. Intronic regions close to the intron–exon boundary, particularly acceptor and donor sites (positions −1, −2, +1, +2), branch-point site, and the polypyrimidine tract, are especially critical for normal splicing. Accurate splicing also depends on the presence of various enhancers and silencers, located in both intronic and exonic regions, as well as additional regulatory elements [5,6,7,8,9,10].

Numerous bioinformatic tools have been developed to predict the potential impact of variants on pre-mRNA splicing, employing diverse algorithmic approaches. Most of these programs focus on the analysis of consensus splice site sequences near intron–exon boundaries, whereas relatively few evaluate the influence of variants on splicing enhancers and silencers located within both exons and introns. Combining multiple prediction tools with different methodologies can improve the reliability of *in silico* assessments, but such predictions require validation through experimental studies [11,12].

The *SLC26A4* gene (solute carrier family 26, member 4, 7q22.3, OMIM #605646, NCBI: NM_000441.2) is one of the key genes involved in the etiology of hearing loss. It is primarily expressed in the inner ear, thyroid gland, and kidneys, and encodes pendrin—a transmembrane transport protein composed of 780 amino acids that functions as a multifunctional exchanger of monovalent anions (I^−^, Cl^−^, HCO_3_^−^, OH^−^, SCN^−^, etc.) [13,14,15,16,17].

Pathogenic variants in *SLC26A4* may cause either nonsyndromic recessive hearing loss (DFNB4, OMIM #600791) or Pendred syndrome (OMIM #274600), the latter characterized by hearing loss combined with thyroid dysfunction. Both conditions are frequently associated with structural abnormalities of the inner ear, the most common of which is an enlarged vestibular aqueduct (EVA). The clinical manifestations of DFNB4 and Pendred syndrome show substantial variability in age of onset, severity, and progression of hearing loss, the extent of inner ear malformations, and the presence and severity of hypothyroidism symptoms [17,18,19,20,21,22,23]. This wide phenotypic spectrum suggests a possible correlation between specific pathogenic *SLC26A4* variants and partial or complete loss of pendrin-mediated anion transport.

Studying the functional consequences of sequence alterations in *SLC26A4* is essential for clarifying genotype–phenotype correlations and for distinguishing truly pathogenic variants from benign changes. Numerous studies have aimed to elucidate these genotype–phenotype correlations in patients with identified biallelic pathogenic *SLC26A4* variants, as well as to investigate the mechanisms of hearing loss in cases where only a single pathogenic *SLC26A4* variant is detected [19,20,21,22,24,25,26,27,28]. An important part of this work is the *in vitro* functional validation of *SLC26A4* variants.

The *SLC26A4* gene is located on chromosome 7 (7:107,660,828–107,717,809; GRCh38 assembly) and consists of 21 exons, including a non-coding first exon. To date, more than 8500 different sequence variations in *SLC26A4* have been reported, of which approximately 600 are associated with hearing loss phenotypes (Deafness Variation Database: https://deafnessvariationdatabase.org/genes/SLC26A4, accessed on 20 July 2025). While most pathogenic variants occur in coding regions and disrupt pendrin structure or function, about 25% are thought to impair splicing. Historically, the splicing effects of these variants were mainly inferred from *in silico* predictions without experimental confirmation. However, growing interest in their experimental verification has led to the application of various *in vitro* splicing assays [29,30,31,32,33,34,35,36,37,38].

In our previous analysis of *SLC26A4* in patients with hearing loss from the Tyva and Altai Republics (Southern Siberia, Russia), we identified a distinctive spectrum of *SLC26A4* variants including well-established pathogenic variants (c.919-2A>G, c.2168A>G) confirmed by functional studies, as well as other known *SLC26A4* variants [39]. We also detected several novel and rare variants located in both intronic and exonic regions of the *SLC26A4* gene. Given the uncertain clinical significance of these variants, the present study aimed to evaluate their potential effects on splicing using a minigene assay.

## 2. Results

### 2.1. In Silico Analysis of the Potential Effects of SLC26A4 Variants on Splicing

Using preliminary bioinformatics analysis with a set of predictive tools capable of assessing the potential impact of variants on splicing, six variants of the *SLC26A4* gene were selected: the already known rare variant c.2034+1G>A (intron 17), whose pathogenicity had not been previously functionally confirmed; the variants c.1545-168A>G (intron 13), c.1708-125T>C (intron 15), c.1708-18T>A (intron 15), and c.1804-31C>T (intron 16), localized in intronic regions outside the canonical splicing sites; and the synonymous exonic variant c.942A>G (p.Ser314=) (exon 8) (Table 1).

According to bioinformatics predictions, the c.2034+1G>A variant results in the loss of the canonical splice donor site through a G to A substitution at position c.2034+1. This change is predicted to lead to the activation of a cryptic donor site and the inclusion of five nucleotides from the intronic region into the transcript, resulting in a frameshift. The rare variant c.1708-18T>A is interpreted as “benign” in the ClinVar database; however, according to bioinformatics predictions, the T to A substitution at this position can cause the loss of the consensus splice acceptor site (according to MaxEntScan) and activation of a cryptic site (according to HSF), which may lead to splicing disruption. For three intronic variants—c.1545-168A>G, c.1708-125T>C and c.1804-31C>T, ambiguous predictive estimates were obtained (Table 1). To evaluate the c.942A>G variant, taking into account its localization (exon 8), several predictive programs were used, including those that are capable of estimating the effects of point mutations affecting potential elements of splicing regulation. Predictive programs showed that the presence of this variant can disrupt splicing by various mechanisms, such as the creation of a new donor splice site (according to HSF) or the loss/disruption of RNA-binding proteins binding sites (according to RBPmap, SpliceAid) (Table 1).

### 2.2. In Vitro Analysis of the SLC26A4 Variants

The splicing analysis was initially performed using the HEK293T cell line and repeated in the SW480 cell line. The experiments were repeated twice for each cell line.

**Figure 1:**
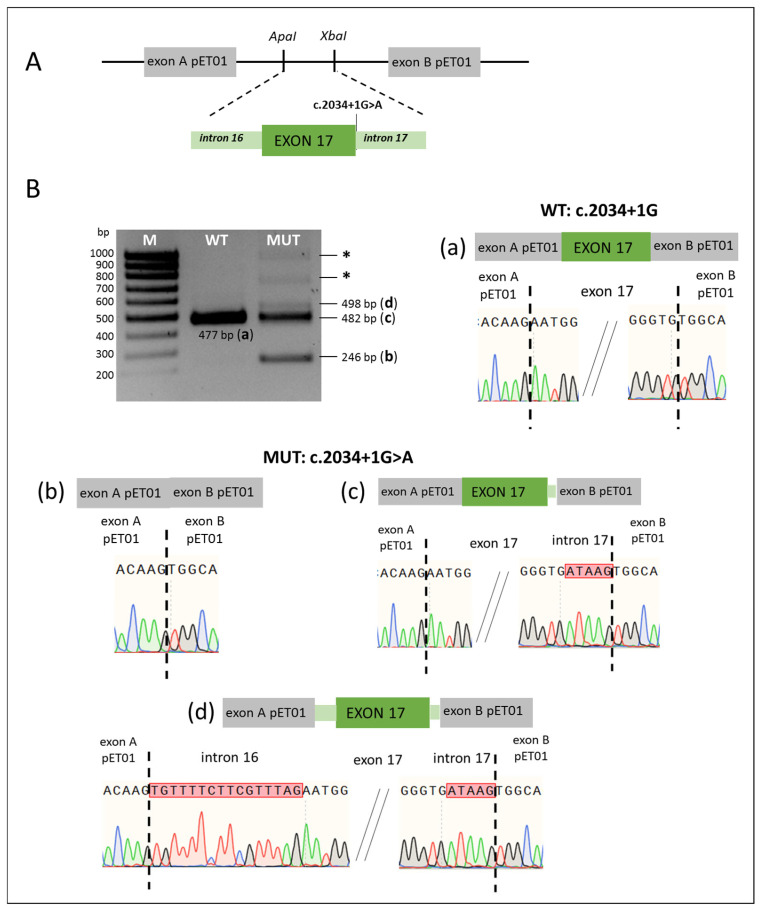
(**A**) Scheme of minigene with the c.2034+1G>A variant. The pET01 vector fragments are colored in gray, the *SLC26A4* gene fragments—in green. *ApaI*, *XbaI*—the recognition sites for restriction endonucleases. (**B**). Results of minigene assay (obtained on the HEK293T cell line) for the c.2034+1G>A variant and the corresponding wild type. The *SLC26A4* transcripts visualized by agarose gel electrophoresis. (**a**–**d**)—schematic representation of the splicing results and partial Sanger sequencing results of PCR products for the wild type (WT) (**a**) and mutant (MUT) (**b**–**d**) minigenes. *—fragments longer than 700 bp correspond to transcripts that include parts of the plasmid sequence. M—100 bp marker. The *SLC26A4* transcripts were also visualized by capillary electrophoresis (Bio-Fragment Analyzer Qsep1) (Supplementary Materials: Figure S1).

**Variant c.2034+1G>A.** The c.2034+1G>A variant (intron 17), located at the canonical position +1 of the donor splice site, was found in one Tuvinian patient in compound heterozygosity with the pathogenic variant c.919-2A>G. This variant is not present in population databases. According to predictive tools, the presence of the c.2034+1G>A variant may disrupt the consensus splice site (Table 1). To investigate the effects of the c.2034+1G>A variant, minigene constructs including exon 17 (carrying either c.2034+1G>A or the wild type sequence) with its flanking intronic regions were created (Figure 1A), and the splicing patterns of the generated transcripts were compared (Figure 1B). In the wild type (WT) minigene, a single fragment of size 477 bp (a) was detected, consistent with normal splicing, in which the intronic regions are completely removed and exon 17 is retained. Correct processing of the mRNA was confirmed by Sanger sequencing of this PCR product. In contrast, multiple bands were detected in the c.2034+1G>A (MUT) minigene, indicating alternative splicing patterns for this mutant *SLC26A4* variant. Analysis of these PCR products by Sanger sequencing revealed the following (Figure 1B(b–d)): the presence of a PCR product of 246 bp (b) reflects a situation where the exon and intron sequences inserted between the pET01 vector exons are completely skipped; the 482 bp fragment (c) reflects a different effect on splicing caused by the c.2034+1G>A variant, which leads to the loss of the canonical wild type donor site, activation of a cryptic donor site, and the inclusion of five nucleotides from intron 17 into the transcript, resulting in a frameshift; the presence of a small amount of the 498 bp fragment (d) represents a splicing pattern in which alteration of both the splice acceptor and splice donor sites results in the inclusion of 16 nucleotides from intron 16 and 5 nucleotides from intron 17. This complex splicing pattern also disrupts the reading frame. As a result of experimental *in vitro* verification, the pathogenic effect of the intronic variant c.2034+1G>A, leading to aberrant splicing, was confirmed.

**Variants c.1708-18T>A and c.1708-125T>C.** Both variants are localized in intron 15 of the *SLC26A4* gene. Each of them was found in individual deaf Tuvinian patients. The c.1708-125T>C variant is novel, while the very rare c.1708-18T>A variant (rs55701254) has previously been reported only in single patients from Tunisia and India [40,41].

**Figure 2:**
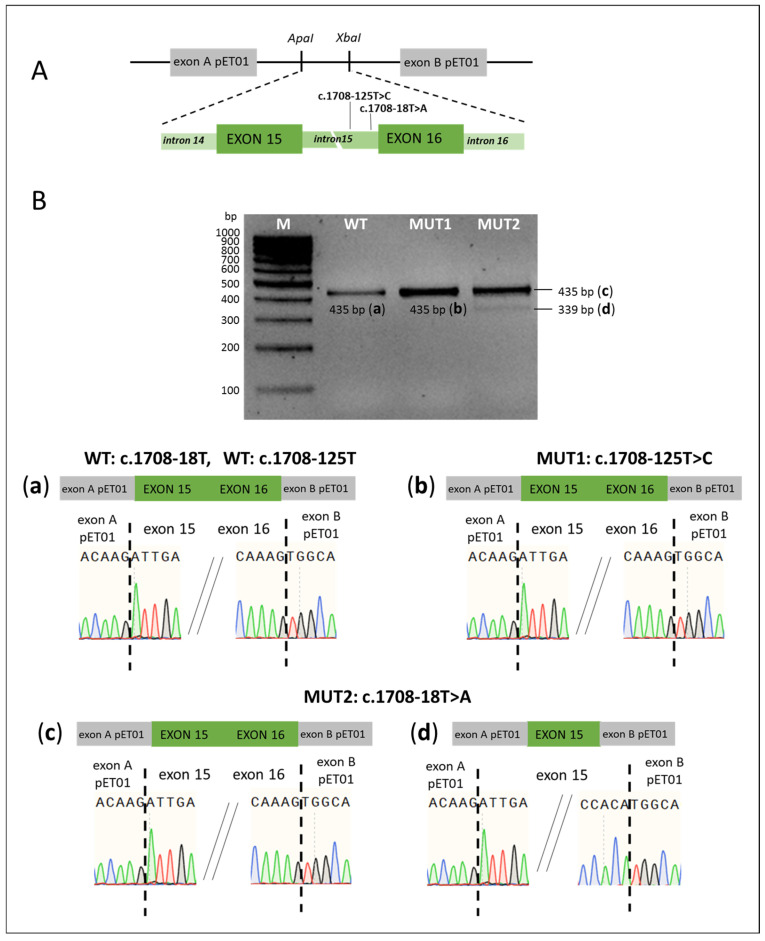
(**A**) Scheme of minigene with variants c.1708-18T>A and c.1708-125T>C. The pET01 vector fragments are colored in gray, the *SLC26A4* gene fragments—in green. *ApaI*, *XbaI*—the recognition sites for restriction endonucleases. (**B**). The results of minigene assay (obtained on the HEK293T cell line) for variants c.1708-18T>A, c.1708-125T>C and the corresponding wild types. The *SLC26A4* transcripts visualized by agarose gel electrophoresis. (**a**–**d**)—schematic representation of the splicing results and partial Sanger sequencing results of PCR products for the wild type (WT) (**a**), for the c.1708-125T>C mutant variant (MUT1) (**b**), and for the c.1708-18T>A mutant variant (MUT2) (**c**,**d**). M—100 bp marker. The *SLC26A4* transcripts were also visualized by capillary electrophoresis (Bio-Fragment Analyzer Qsep1) (Supplementary Materials: Figure S2).

Although c.1708-18T>A variant is classified as “benign” in the ClinVar database, bioinformatics analysis predicts that the T to A substitution at position c.1708-18 may disrupt the consensus splice acceptor site (according to MaxEnt) and activate a cryptic site (according to HSF), likely leading to disruption of splicing (Table 1). To study the c.1708-18T>A and c.1708-125T>C variants, a total of three minigene constructs were generated, each including the *SLC26A4* genomic region encompassing exons 15 and 16, the entire intron 15 (925 bp), and the corresponding wild-type or mutant variant with flanking intronic sequences (Figure 2A). Analysis of PCR products in minigenes with the c.1708-125T>C variant and the corresponding wild type variant revealed no differences in splicing patterns, as in both cases the same PCR product (435 bp) was detected (Figure 2B(a,b)), indicating no effect of the c.1708-125T>C variant on splicing. Analysis of minigene containing the c.1708-18T>A variant revealed two fragments: (c) of size 435 bp corresponding to a normal splicing pattern and (d) of size 339 bp showing skipping of exon 16, apparently due to disruption of the acceptor site (Figure 2B(c,d)). It should be noted that exon 16 skipping in the presence of the c.1708-18T>A variant was observed only in a small proportion of transcripts.

**Figure 3:**
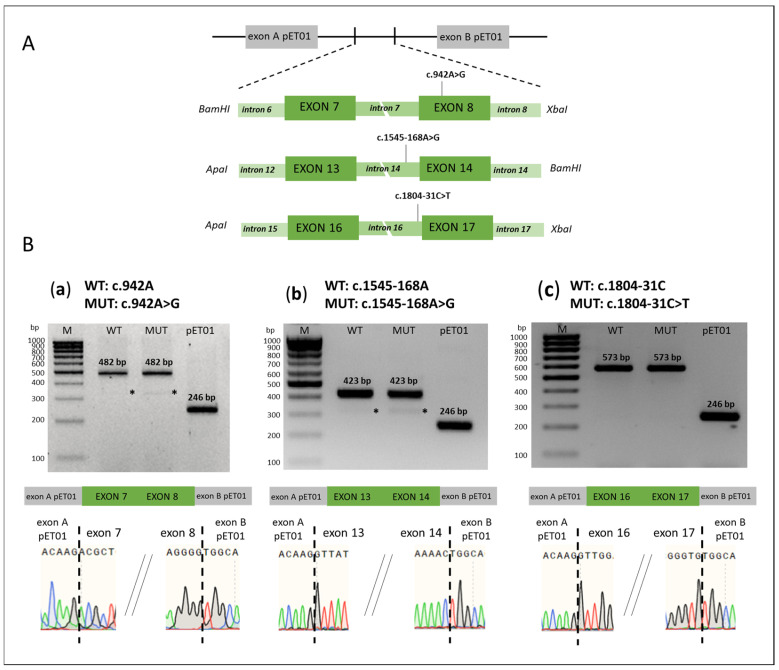
(**A**) Schemes of minigenes with variants c.942A>G, c.1545-168A>G, and c.1804-31C>T. The pET01 vector fragments are colored in gray, the *SLC26A4* gene fragments—in green. *ApaI*, *XbaI*, *BamHI*—the recognition sites for restriction endonucleases. (**B**). The results of minigene assay (obtained on the HEK293T cell line) for variants c.942A>G, c.1545-168A>G, c.1804-31C>T and the corresponding wild types: schematic representation of the splicing results, visualization by agarose gel electrophoresis, and partial Sanger sequencing results of PCR products. (**a**) WT: c.942A and MUT: c.942A>G; (**b**) WT: c.1545-168A and MUT: c.1545-168A>G; (**c**) WT: c.1804-31C and MUT: c.1804-31C>T. WT—wild type; MUT—mutant variant; pET01—pET01 vector without insertion; *—PCR products resulting from the activation of cryptic splicing sites in the genetic construct used. M—100 bp marker. The *SLC26A4* transcripts were also visualized by capillary electrophoresis (Bio-Fragment Analyzer Qsep1) (Supplementary Materials: Figure S3).

**Variants c.1545-168A>G, c.1804-31C>T, and c.942A>G.** The c.1545-168A>G variant is located in intron 14, c.1804-31C>T variant in intron 16, and c.942A>G variant in exon 8. Ambiguous predictive estimations were obtained for all of these *SLC26A4* variants (Table 1). For the functional analysis of these variants, we generated a total of six minigene constructs, including the corresponding *SLC26A4* genomic regions (exon—intron—exon) carrying the mutant or wild type variants with flanking intronic regions (Figure 3A). Functional analysis did not reveal any effect of these variants on splicing (Figure 3B).

All results of the splicing analysis obtained with primary transfection of all minigenes in the HEK293T cell line were identical to those obtained from transfection in the SW480 cell line (Supplementary Materials: Figure S4).

## 3. Discussion

In this study, we analyzed the effects of several novel and rare variants of the *SLC26A4* gene on splicing. Based on predictions from bioinformatic tools, six variants identified in patients with hearing loss from the Altai and Tyva Republics (Southern Siberia, Russia) were selected for *in vitro* analysis: one canonical donor splice site variant (c.2034+1G>A), four non-canonical intronic variants (c.1545-168A>G, c.1708-18T>A, c.1708-125T>C, and c.1804-31C>T), and one synonymous exonic variant, c.942A>G (p.Ser314=).

To date, 143 pathogenic *SLC26A4* variants associated with abnormal splicing, including 91 intronic and 52 exonic variants, are reported in the Deafness Variation Database (https://deafnessvariationdatabase.org/genes/SLC26A4, accessed on 20 July 2025). Historically, deleterious effects of these variants on splicing were inferred mostly from *in silico* predictions without experimental validation. However, interest in experimentally confirming their impact using *in vitro* assays has recently increased. Table 2 summarizes all known in our opinion experimental studies to date [29,30,31,32,33,34,35,36,37,38,42,43,44,45,46,47]. We also provide corresponding *in silico* predictions for these variants. The potential impact on splicing was assessed using various computational tools, which differed across studies. In most cases, the variants predicted as “pathogenic” (i.e., with potential damaging effect on splicing) were selected for analysis; however, several variants had ambiguous predictions.

Among intronic *SLC26A4* variants, the most common (~66%, 60/91) are classical splicing variants that disrupt canonical splicing acceptor or donor sites (positions −1, −2 or +1, +2). These variants are typically predicted to abolish normal splicing and result in exon skipping or alternative splicing. To date, experimental splicing analyses have been reported for only six such canonical variants: c.601-1G>A [31], c.919-2A>G [31,45], c.1002-1G>T [33], c.1149+1G>A [37], c.1545-2A>G, and c.1614+1G>C [33] (Table 2). These studies have revealed different splicing patterns for some of them. For example, c.919-2A>G, a frequent variant among Asian patients, located in the canonical splice acceptor site in intron 7, causes complete exon 8 skipping and introduces a premature stop codon at position 311, leading to a truncated pendrin protein [45]. Wasano et al. (2020) showed that this variant may also lead to skipping of both exons 7 and 8 [31]. They also demonstrated that variant c.601-1G>A disrupts the splice acceptor site of intron 5, causing partial loss of exon 5 [31]. Albader et al. (2022) showed that the c.1545-2A>G variant produces multiple aberrant transcripts, including partial exon 14 skipping due to activation of cryptic splice sites and complete exon 14 skipping [33].

In our study, we investigated the rare canonical donor splice site variant c.2034+1G>A (intron 17). We demonstrated that this variant can cause three distinct splicing defects: (1) complete skipping of exon 17, resulting in deletion of 77 amino acids; (2) partial retention (5 nucleotides) of intron 17 due to activation of a cryptic donor site, leading to a frameshift and premature stop at position 690; (3) retention of 16 nucleotides from intron 16 and 5 nucleotides from intron 17 due to activation of both cryptic donor and acceptor sites, causing a frameshift and premature stop at position 610.

Another subset of intronic variants presumed to affect splicing includes those located outside of canonical splice sites—typically within about 50 nucleotides of exon–intron junctions. This is likely because these regions are more likely to be covered in standard diagnostic testing, which targets exons (20 exons) and nearby intronic sequences. An increasing number of studies are focusing on the investigation of these variants in the *SLC26A4* gene [29,30,31,32,33,36,37,42,43,44] (Table 2). Notably, in almost half of these cases (12 of 28), including two variants from our study (c.1708-18T>A and c.1804-31C>T), there were inconsistencies between *in silico* predictions and functional assay results, highlighting a weak correlation between bioinformatics predictions and the experimental data. Variant c.1708-18T>A only partially matched the predicted estimates (Table 1), as most transcripts exhibited normal splicing, although a small proportion skipped exon 16. Exon 16 skipping results in a deletion of 32 amino acids located in the STAS (Sulfate Transporter and AntiSigma factor antagonist) domain (aa 535–729) (https://www.ebi.ac.uk/interpro/entry/pfam/PF01740/, accessed on 25 July 2025). This domain is a functionally important region of the pendrin protein, as well as other SLC26 protein family members, as it appears to be involved in nucleotide binding and/or interactions with other proteins. For the c.1804-31C>T variant, no splicing disruption was observed.

A distinct category includes deep intronic variants (>100 bp from exon–intron boundaries). These variants are likely to lead to pseudo-exon inclusion due to activation of non-canonical splice sites or changes in splicing regulatory elements, and also disrupt regulatory transcriptional motifs and non-coding RNA genes [9,10]. Functional evidence for deleterious effects has been reported for two deep intronic variants, c.304+941C>T [35] and c.1707+94C>T [33], while our study found no effect on splicing for c.1545-168A>G or c.1708-125T>C (Table 2).

Finally, exonic variants (including synonymous substitutions) can influence splicing by creating novel splice sites, activating cryptic sites, or disrupting exonic splicing enhancers (ESEs), potentially resulting in partial or complete exon skipping. Growing evidence supports the regulatory role of synonymous variants in post-transcriptional processing [5,9,10,48,49]. Table 2 summarizes both *in silico* and *in vitro* data for exonic variants [30,31,33,34,38,46,47]. In our study, we found that the synonymous variant c.942A>G (p.Ser314=), despite predicted splicing disruption, does not affect splicing. In our recent work [38], the c.1545T>G variant (located at the first nucleotide of exon 14) was shown to have no splicing impact despite ambiguous predictions. Overall, for 10 out of 14 exonic variants (Table 2), bioinformatic predictions did not match experimental outcomes, underscoring the limitations of *in silico* tools in assessing splicing consequences of exonic variants.

## 4. Materials and Methods

### 4.1. In Silico Analysis of the SLC26A4 Variants

*In silico* analysis was performed using a set of bioinformatics predictive tools to assess the potential effects of genetic variants on splicing: for intronic variants—Human Splicing Finder (including the MaxEnt algorithm, https://genomnis.com/hsf, accessed on 20 May 2025), SpliceAI (https://spliceailookup.broadinstitute.org/, accessed on 20 May 2025), CADD (https://cadd.gs.washington.edu/, accessed on 20 May 2025), FATHMM-MKL, and FATHMM-XF Non-Coding Variants (https://fathmm.biocompute.org.uk/, accessed on 20 May 2025); for exonic variants—Human Splicing Finder (including the MaxEnt algorithm, https://genomnis.com/hsf, accessed on 20 May 2025), SpliceAI (https://spliceailookup.broadinstitute.org/, accessed on 20 May 2025), EX-SKIP (https://ex-skip.img.cas.cz/, accessed on 20 May 2025), SpliceAid (http://www.introni.it/splicing.html), and RBPmap (https://rbpmap.technion.ac.il/, accessed on 20 May 2025).

### 4.2. Generation of Minigene Constructs

To create minigene constructs, genomic regions of the *SLC26A4* gene (NG_008489.1) containing either one or two exons, as well as approximately 170 bp of flanking intronic regions, were selected. In total, the following minigenes were generated: exons 7–8 (with the c.942A>G or wild type variants), exons 13–14 (with the c.1545-168A>G, c.1545T>G or wild type variants), exons 15–16 (with the c.1708-18T>A, c.1708-125T>C or wild type variants), exons 16–17 (with c.1804-31C>T or wild type variants), and exon 17 (with c.2034+1G>A or wild type variants).

To amplify the *SLC26A4* gene fragments, primers were designed using the Primer3Plus program (https://www.primer3plus.com/index.html, accessed on 1 May 2025). These primers included recognition sites for the restriction endonucleases *BamHI*, *XbaI* or *ApaI* at their 5’ends (Table 3). The amplified fragments were cloned into the Exontrap pET01 plasmid vector (MoBiTec GmbH, Goettingen, Germany) at the corresponding restriction sites using T4 DNA ligase (SibEnzyme, Novosibirsk, Russia).

Genomic DNA with *SLC26A4* variants c.1545-168A>G, c.1708-18T>A, c.1708-125T>C, and c.2034+1G>A, as well as DNA without changes at the corresponding nucleotide positions, was obtained from patients with hearing loss in our previous study [39]. Minigenes with the c.1804-31C>T and c.942A>G variants were generated by site-directed mutagenesis using specific primers (Table 3) and the KLD Enzyme Mix kit (New England Biolabs, Ipswich, USA). The obtained minigene constructs were further sequenced to verify the absence of additional sequence changes.

### 4.3. Cell Lines

Splicing patterns were initially analyzed using the HEK293T (human embryonic kidney) cell line, and experiments were repeated in the SW480 (human colorectal adenocarcinoma) cell line. Both cell lines (HEK293T and SW480) were acquired from the company PrimeBioMed (Moscow, Russia). The cell lines were cultured in DMEM/F12 medium (Biolot, Saint-Petersburg, Russia) supplemented with 10% fetal bovine serum (Capricorn Scientific, Marburg, Germany) and 2 mM penicillin/streptomycin antibiotic mixture (Capricorn Scientific, Marburg, Germany) at 37 °C in 5% CO_2_ incubator. The cell lines were screened for mycoplasma contamination using the MycoReport kit (Evrogen, Moscow, Russia).

### 4.4. Minigene Assay

Cell lines (HEK293T or SW480) were transfected with either mutant or wild-type minigenes using Lipofectamine 3000 (Life Technologies/Thermo Fisher Scientific, Waltham, MA, USA) according to the manufacturer’s instructions. Total RNA was isolated from the cells 48 h after transfection using the RNA solo kit (Evrogen, Moscow, Russia). The isolated RNA was treated with rDSN DNase (RNA solo kit), and cDNA was synthesized using the MMLV RT kit (Evrogen, Moscow, Russia) with a primer (cDNA primer 1) specific to the plasmid region of the pET01 vector (Table 3). Subsequently, using the obtained cDNA as a template, the fragments corresponding to the minigenes were amplified with primers (PCR primer 02 and PCR primer 03) specific to the exons of the pET01 vector (Table 3). The PCR products were analyzed by 3% agarose gel electrophoresis and by capillary electrophoresis using the automated Bio-Fragment Analyzer Qsep1 (BiOptic, New Taipei City, Taiwan), followed by Sanger sequencing.

## 5. Conclusions

Several novel and rare *SLC26A4* variants, predicted *in silico* to potentially disrupt splicing, were evaluated using a minigene assay. The *in vitro* results were only partially consistent with the *in silico* predictions. These findings indicate that reliance solely on computational predictions is insufficient for clinical or diagnostic decision making; experimental *in vitro* validation is essential to confirm predicted functional effects.

## Figures and Tables

**Table 1:** *In silico* analysis of *SLC26A4* variants.

**Intronic Variants**						
**Variant** **(Location in the *SLC26A4* Gene)**	**Position** **GRCh38.p14: chr 7,** **NC_000007.14:**	**Human Splicing Finder (HSF)**	**SpliceAI**	**CADD**	**FATHMM-MKL Non-Coding Score**	**FATHM-XF** **Non-Coding Score**
c.2034+1G>A rs759683649 (intron 17)	g.107702058G>A	New Donor splice site: Activation of a cryptic Donor site. Potential alteration of splicing (HSF) Broken WT Donor Site: Alteration of the WT Donor site, most probably affecting splicing (HSF) Broken WT Donor Site: Alteration of the WT Donor site, most probably affecting splicing (MaxEnt)	Donor Loss 1.0 Donor Gain 0.81	35	0.99333 pathogenic	0.991095 pathogenic
c.1545-168A>G * rs1791783004 (intron 13)	g.107697874A>G	Alteration of auxiliary sequences: Significant alteration of ESE/ESS motifs ratio (5)	0.0	18.2	0.93758 pathogenic	0.373789 benign
c.1708-125T>C (intron 15)	g.107700976T>C	Alteration of auxiliary sequences: Significant alteration of ESE/ESS motifs ratio (6)	0.0	3.529	0.17111 benign	0.048611 benign
c.1708-18T>A rs55701254 (intron 15)	g.10770108 3T>A	New Acceptor splice site: Activation of a cryptic Acceptor site. Potential alteration of splicing (HSF) Broken WT Acceptor Site: Alteration of the WT Acceptor site, most probably affecting splicing (MaxEnt)	Acceptor Loss 0.01	21.2	0.98823 pathogenic	0.565967 pathogenic
c.1804-31C>T (intron 16)	g.107701796C>T	Broken WT Branch Point: Alteration of the WT Branch Point, may affect splicing	Acceptor Gain 0.04	4.452	0.19947 benign	0.073999 benign
**Exonic Variant**						
**Variant** **(Location in the *SLC26A4* gene)**	**Position** **GRCh38.p14: chr 7,** **NC_000007.14:**	**Human Splicing Finder (HSF)**	**SpliceAI**	**RBPmap**	**SpliceAid**	**EX-SKIP**
c.942A>G * (p.Ser314=) rs2535310634 (exon 8)	g.107683478A>G	New Donor splice site: Activation of a cryptic Donor site. Potential alteration of splicing (HSF)	Donor Loss 0.02	Loss or disruption of binding sites of RNA-binding proteins	Loss or disruption of binding sites of RNA-binding proteins	Allele c.942A>G has a higher chance of exon skipping than allele c.942A

* novel variants at the time of study. FATHMM–MKL and FATHM–XF: scores above 0.5 are predicted to be pathogenic, while those below 0.5 are predicted to be benign. CADD scores greater than 20 are considered to be pathogenic. SpliceAI: variants with scores greater than 0.80 are likely to cause splicing changes.

**Table 2:** *In vitro* validation of effects of the *SLC26A4* gene variants on splicing.

*SLC26A4* Variant	Location	*In Silico* Predictions for Splicing Defect	*In Vitro* Analysis	Reference
**Canonical Splice Acceptor and Donor Sites (Positions −1, −2, +1, +2)**				
c.601-1G>A	CSAS (intron 5)	Pathogenic	A partial loss of exon 5	[31]
c.919-2A>G	CSAS (intron 7)	Pathogenic	Splicing errors: (1) exon 8 skipping [45]; (2) exons 7 and 8 skipping (60% transcripts), exon 8 skipping (40% transcripts) [31]	[31,45]
c.1002-1G>T	CSAS (intron 8)	Pathogenic	Exon 9 skipping	[33]
c.1149+1G>A	CSDS (intron 9)	Pathogenic	Exon 9 skipping	[37]
c.1545-2A>G	CSAS (intron 13)	Pathogenic	Splicing errors: (1) exon 14 skipping (~80%); (2) 9 bp (~10%) and 24 bp (~10%) deletions of exon 14, respectively, due to activation of cryptic splice acceptor site	[33]
c.1614+1G>C	CSDS (intron 14)	Pathogenic	Exon 14 skipping	[33]
c.2034+1G>A	CSDS (intron 17)	Pathogenic	Splicing errors: (1) exon 17 skipping; (2) 5 bp retention of intron 17 due to activation of a cryptic donor site in intron 17; (3) 16 bp retention of intron 16 and 5 bp retention of intron 17 due to activation of a cryptic donor and acceptor sites	**This study**
**Intronic variants outside canonical splice sites** (**within 50 nucleotides from intron–exon boundaries)**				
c.165-5G>A ^a^	NSAS (intron 2)	Pathogenic	No splicing error	[33]
c.165-13T>G ^a^	NSAS (intron 2)	Pathogenic	No splicing error	[33]
c.415+4A>G ^a^	NSDS (intron 4)	Pathogenic	No splicing error [30]; exon 4 skipping [33]	[30,33]
c.415+7A>G	NSDS (intron 4)	Pathogenic	Activation of a cryptic splice donor site in intron 4 leading to 6 bp retention of intron 4	[33,42]
c.765+3A>C	NSDS (intron 6)	Pathogenic	Exon 6 skipping	[33]
c.765+3A>T	NSDS (intron 6)	Pathogenic	Exon 6 skipping	[30,33]
c.765+4A>C ^a^	NSDS (intron 6)	Pathogenic	No splicing error	[33]
c.765+4A>G	NSDS (intron 6)	Pathogenic	Exon 6 skipping	[36]
c.1001+4A>G	NSDS (intron 8)	Pathogenic	Activation of a cryptic splice donor site in intron 8 leading to 40 bp retention of intron 8	[30,33]
c.1001+5G>C	NSDS (intron 8)	Pathogenic	Activation of a cryptic splice donor site in intron 8 leading to 40 bp retention of intron 8	[30,33]
c.1001+5G>T	NSDS (intron 8)	Pathogenic	Activation of a cryptic splice donor site in intron 8 leading to 40 bp retention of intron 8	[30,33]
c.1001+30A>G ^a^	NSDS (intron 8)	Ambiguous	No splicing error	[33]
c.1002-4C>G	NSAS (intron 8)	Pathogenic	Exon 9 skipping	[33,43]
c.1002-8C>G	NSAS (intron 8)	Pathogenic	Exon 9 skipping	[33]
c.1002-9A>C ^a^	NSAS (intron 8)	Pathogenic	No splicing error	[29,33]
c.1002-9A>G	NSAS (intron 8)	Pathogenic	Exon 9 skipping	[33]
c.1149+3A>G	NSDS (intron 9)	Pathogenic	Exon 9 skipping	[30,44]
c.1341+3A>C	NSDS (intron 11)	Pathogenic	Exon 11 skipping	[30]
c.1544+5G>A	NSDS (intron 13)	Pathogenic	Exon 13 skipping	[30,33]
c.1544+9C>G ^a^	NSDS (intron 13)	Ambiguous	Activation of a cryptic splice donor site in intron 13, leading to 9 bp retention of intron 13	[33]
c.1544+9C>T ^a^	NSDS (intron 13)	Ambiguous	No splicing error	[29,33]
c.1545-5T>G ^a^	NSAS (intron 13)	Pathogenic	No splicing error	[29,33]
c.1614+5G>A	NSDS (intron 14)	Pathogenic	Exon 14 skipping	[32]
c.1614+7A>G ^a^	NSDS (intron 14)	Ambiguous	No splicing error	[33]
c.1707+5G>A	NSDS (intron 15)	Pathogenic	A complete loss of exon 15	[30,31]
c.1708-18T>A ^a^	NSAS (intron 15)	Pathogenic	(1) no splicing error; (2) exon 16 skipping in a small proportion of transcripts	**This study**
c.1804-31C>T ^a^	NSAS (intron 16)	Ambiguous	No splicing error	**This study**
c.2089+3A>T	NSDS (intron 18)	Pathogenic	Exon 18 skipping	[37]
**Deep intronic variants**				
c.304+941C>T ^a^	DIV (intron 3)	Ambiguous	Activation of a cryptic donor and acceptor sites leading to 126 bp retention of intron 3	[35]
c.1545-168A>G ^a^	DIV (intron 13)	Ambiguous	No splicing error	**This study**
c.1707+94C>T ^a^	DIV (intron 15)	Benign	Skipping of exons 15 and 16	[33]
c.1708-125T>C ^a^	DIV (intron 15)	Ambiguous	No splicing error	**This study**
**Exonic variants**				
c.162C>T (p.Cys54=)	Exon 2	Pathogenic	Splicing errors: (1) exon 2 skipping; (2) 4 bp deletion of exon 2 due to activation of cryptic splice donor site	[34]
c.304G>A (p.Gly102Arg)	Last nucleotide of exon 3	Pathogenic	Exon 3 skipping	[30]
c.392G>T ^a^ (p.Gly131Val)	Exon 4	Pathogenic	(1) exon 4 skipping; (2) no splicing error	[31]
c.918G>A ^a^ (p.Val306=)	Last nucleotide of exon 7	Ambiguous	Exon 7 skipping	[33]
c.942A>G ^a^ (p.Ser314=)	Exon 8	Pathogenic	No splicing error	**This study**
c.1001G>T (p.Gly334Val)	Last nucleotide of exon 8	Pathogenic	Activation of a cryptic splice donor site in intron 8 leading to 40 bp retention of intron 8	[30,31,33,46]
c.1261C>A ^a^ (p.Gln421Lys)	Exon 10	Pathogenic	No splicing error	[30]
c.1262A>C ^a^ (p.Gln421Pro)	Exon 10	Pathogenic	No splicing error	[30]
c.1262A>G ^a^ (p.Gln421Arg)	Exon 10	Pathogenic	No splicing error	[30]
c.1262A>T ^a^ (p.Gln421Leu)	Exon 10	Pathogenic	No splicing error	[30]
c.1544T>G ^a^ (p.Phe515Cys)	Last nucleotide of exon 13	Pathogenic	No splicing error	[30]
c.1545T>G ^a^ (p.Phe515Leu)	First nucleotide of exon 14	Ambiguous	No splicing error	[38]
c.1614C>T ^a^ (p.Asn538=)	Last nucleotide of exon 14	Ambiguous	No splicing error	[33]
c.1803G>A (p.Lys601=)	Last nucleotide of exon 16	Pathogenic	Exon 16 skipping	[30,47]

^a^—the contradictions between predictive estimations and the results of functional *in vitro* analysis of the *SLC26A4* variants. Ambiguous—the ambiguous predictions obtained using different sets of predictive tools. CSAS—canonical splice acceptor site; CSDS—canonical splice donor site; NSAS—noncanonical splice acceptor site; NSDS—noncanonical splice donor site; DIV—deep intronic variant. Variants investigated in this study are shown in gray.

**Table 3:** Primer sequences and corresponding PCR products.

**Fragments**	**Primer Sequences**	**Product Size, bp**	**Restriction Site**	**Reference**
**Primers used to amplify of the *SLC26A4* gene fragments**				
Exons 7–8	F: GAATGGGATCCTCACCCAGTTTTTCCTTTCC	867	*BamHI*	This study
	R: AAGTTTCTAGAAGGACTCTGGTGTTAACCGTA		*XbaI*	
Exons 13–14	F: TATCATGGGCCCACTGCACTCC	2516	*ApaI*	[38]
	R: GCCGGATCCGTCAGAAGGTGCACT		*BamHI*	
Exons 15–16	F: TACTGGGCCCTGCGCAACAGAGTGAAA	1486	*ApaI*	This study
	R:ATTTTCTAGACTTCCACTCCCGCTTGCCTAT		*XbaI*	
Exons 16–17	F: TTCTGGGCCCGAGTAGGGTAGCCTGGG	1322	*ApaI*	This study
	R:CTCCTCTAGATAGTCTGGCTCCAGAGCCT		*XbaI*	
Exon 17	F: GTTTGGGCCCTAGACAACATCAAAGTTT	587	*ApaI*	This study
	R: CTCCTCTAGATAGTCTGGCTCCAGAGCCT		*XbaI*	
**Primers used for site-directed mutagenesis of the *SLC26A4* variants**				
c.1804-31C>T	F: TTTGA**T**AATTAAGTTGACAGTGTTTTC	-	-	This study
	R: GATTTGAAATCTTTCAACCATTCATA			
c.942A>G	F: CATTTC**G**TATGGAGCCAACCTGG	-	-	This study
	R: GCAGTAGCAATTATCGTCTG			
**Primers specific to the plasmid region of the pET01 vector**				
cDNA	cDNA primer 1: GATCCACGATGC	-	-	https://www.mobitec.com/
pET01exons	PCR primer 02: GATGGATCCGCTTCCTGCCCC	246	*BamHI*	https://www.mobitec.com/
	PCR primer 03: CTCCCGGGCCACCTCCAGTGCC		*SmaI*	

The 5’ ends containing recognition sites for restriction endonucleases are underlined; modified nucleotides in primers for site-directed mutagenesis are shown in bold.

## Data Availability

The data presented in this study are available in the article and in the Supplementary Materials.

## Supplementary Materials

The following supporting information can be downloaded at: https://www.mdpi.com/article/10.3390/ijms262110732/s1.
