# Supplementary material for: Assessing the Functional Significance of Novel and Rare Variants of the SLC26A4 Gene Found in Patients with Hearing Loss by Minigene Assay
Source: Int J Mol Sci. 2025 Nov 4;26(21):10732. doi: 10.3390/ijms262110732 (PMC12610559; doi:10.3390/ijms262110732)
Supplement: Supplementary file 1 [file ijms-26-10732-s001.zip › ijms-3869023-supplementary.pdf]

WT: c.2034+1G

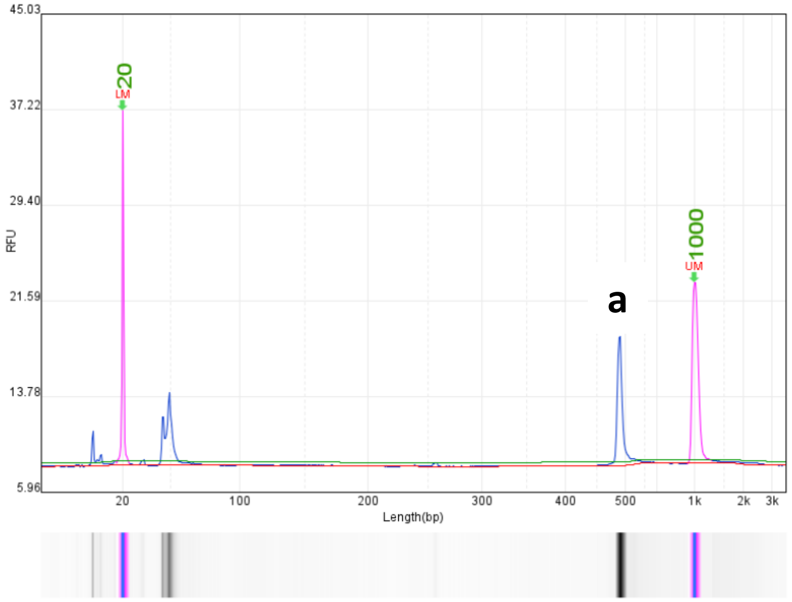

No	Time (sec.)	RFU	bp	Concn. (ng/µl)	PeakArea	PeakStart (sec.)	PeakEnd (sec.)
1	99.88	28.89	20	N/A	296,810	98.64	101.24
2	198.96	10.52	490	0.65	310,060	197.64	201.60
3	213.92	14.76	1,000	N/A	542,981	212.64	217.48

MUT: c.2034+1G>A

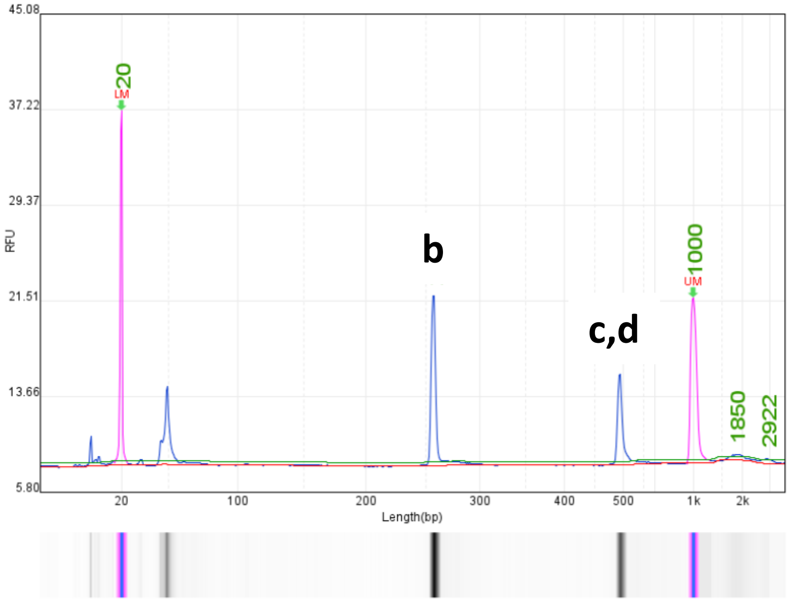

No	Time (sec.)	RFU	bp	Concn. (ng/µl)	PeakArea	PeakStart (sec.)	PeakEnd (sec.)
1	100.44	29.06	20	N/A	300,139	99.08	101.80
2	163.28	13.98	257	0.90	355,807	161.92	164.92
3	200.76	7.49	493	0.48	233,643	199.44	204.12
4	215.52	13.59	1,000	N/A	499,741	214.24	219.12
5	224.20	0.46	1,850	N/A	7,313	223.76	224.40
6	230.56	0.36	2,922	N/A	11,916	230.40	233.32

Figure S1. Capillary electrophoresis (Bio-Fragment Analyzer Qsep1) for PCR products for the wild type minigene (WT) (**a**) and mutant minigene (MUT) with the c.2034+1G>A variant (**b**–**d**). The *SLC26A4* transcripts are visualized by the blue peaks. The PCR products (**c**) and (**d**) are not separated by capillary electrophoresis due to limitations of instrument resolution and the small amount of fragment (**d**). The peaks corresponding the fragments higher than 1000 bp correspond to transcripts that include parts of the plasmid sequence. LM, UM – markers (20 bp and 1000 bp, respectively).

WT: c.1708-18T, WT: c.1708-125T **MUT1: c.1708-125T>C** **MUT2: c.1708-18T>A**

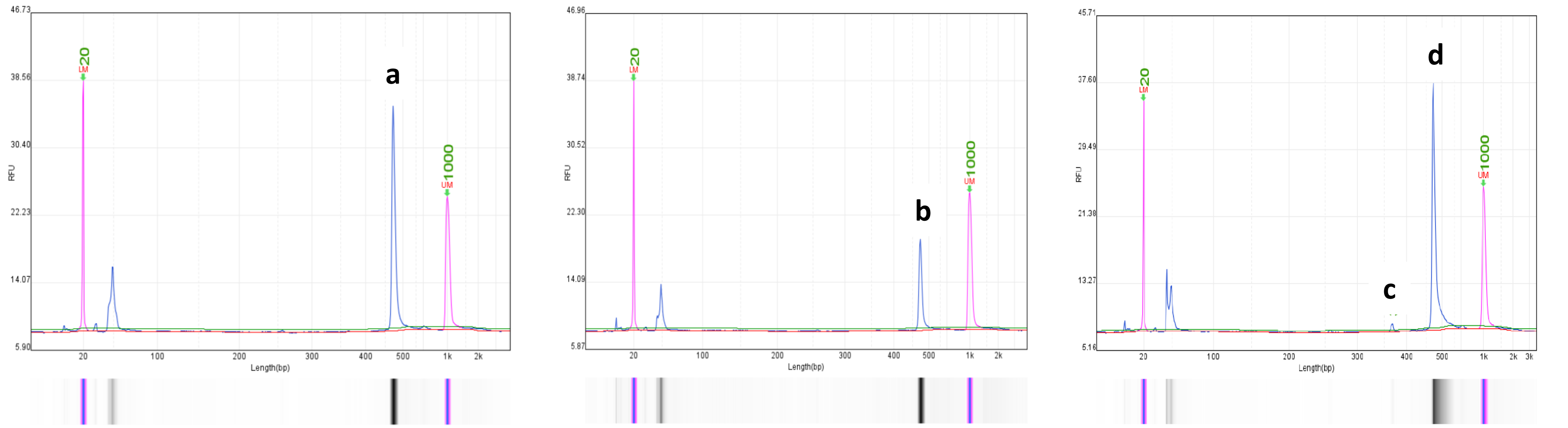

WT:

No	Time (sec.)	RFU	bp	Concn. (ng/µl)	PeakArea	PeakStart (sec.)	PeakEnd (sec.)
1	93.48	0.79	<LM	N/A	8,977	93.00	93.84
2	94.32	0.44	<LM	N/A	11,100	93.84	95.32
3	97.84	0.32	<LM	N/A	4,408	97.16	98.12
4	99.40	30.22	20	N/A	308,269	98.12	100.76
5	195.84	27.21	472	1.95	913,620	194.28	199.68
6	212.68	16.00	1,000	N/A	605,688	211.48	217.36

MUT1:

No	Time (sec.)	RFU	bp	Concn. (ng/µl)	PeakArea	PeakStart (sec.)	PeakEnd (sec.)
1	99.36	30.43	20	N/A	308,417	98.00	100.72
2	195.84	11.19	475	0.78	366,359	194.28	200.16
3	212.40	16.74	1,000	N/A	638,334	211.08	216.88

MUT2:

No	Time (sec.)	RFU	bp	Concn. (ng/µl)	PeakArea	PeakStart (sec.)	PeakEnd (sec.)
1	103.20	27.89	20	N/A	291,364	101.72	104.60
2	190.12	0.95	368	0.06	27,900	188.76	191.12
3	204.36	29.87	472	2.39	1,173,498	202.52	213.20
4	222.04	17.11	1,000	N/A	637,308	220.52	225.56

Figure S2. Capillary electrophoresis (Bio-Fragment Analyzer Qsep1) for PCR products for the wild type minigene (WT) (**a**), mutant minigene with variant c.1708-125T>C (MUT1) (**b**), and mutant minigene with variant c.1708-18T>A (MUT2) (**c**,**d**). The *SLC26A4* transcripts are visualized by the blue peaks. LM, UM – markers (20 bp and 1000 bp, respectively).

WT: c.942A

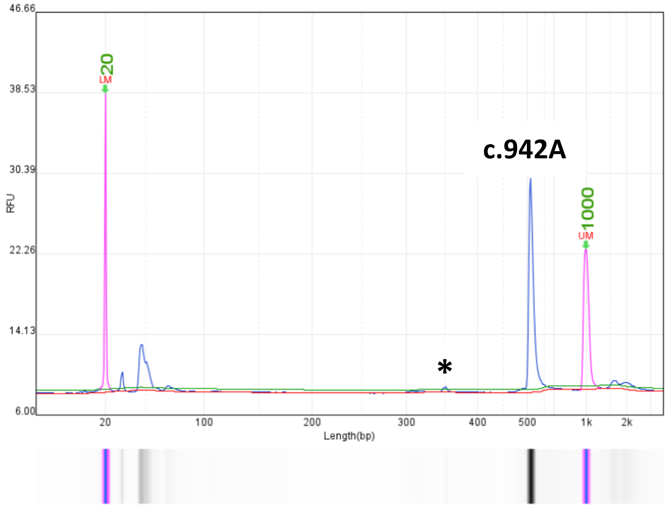

No	Time (sec.)	RFU	bp	Concn. (ng/μl)	PeakArea	PeakStart (sec.)	PeakEnd (sec.)
1	98.08	30.15	20	N/A	308,426	96.80	99.44
2	177.32	0.53	351	0.04	16,668	175.60	178.64
3	197.12	21.55	507	1.52	725,121	195.96	201.92
4	210.00	14.10	1,000	N/A	523,381	208.76	213.20

MUT: c.942A>G

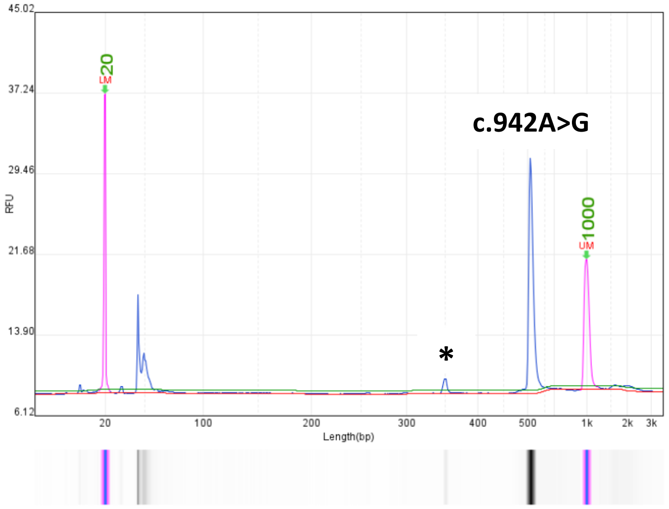

No	Time (sec.)	RFU	bp	Concn. (ng/μl)	PeakArea	PeakStart (sec.)	PeakEnd (sec.)
1	98.24	28.80	20	N/A	289,603	96.96	99.56
2	177.16	1.41	350	0.09	39,293	175.96	178.32
3	196.92	22.66	506	1.47	698,382	195.68	201.00
4	209.88	12.49	1,000	N/A	449,285	208.64	212.80

WT: c.1545-168A

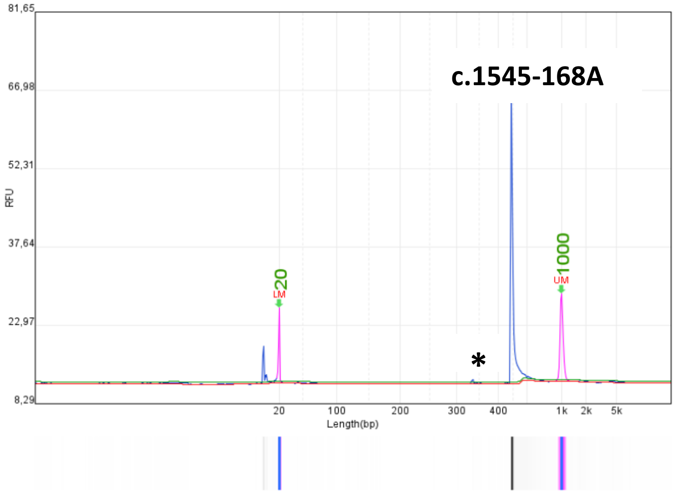

No	Time (sec.)	RFU	bp	Concn. (ng/μl)	PeakArea	PeakStart (sec.)	PeakEnd (sec.)
1	107,76	7,04	<LM	N/A	73 630	106,92	108,20
2	108,68	1,64	<LM	N/A	25 106	108,20	108,96
3	109,20	1,75	<LM	N/A	30 214	108,96	110,40
4	113,12	0,50	<LM	N/A	6 304	112,60	113,28
5	114,08	0,95	<LM	N/A	15 079	113,28	114,16
6	115,24	14,13	20	N/A	185 894	114,16	116,28
7	206,44	0,79	336	0,04	19 271	205,16	207,28
8	224,64	54,74	444	3,41	1 823 347	223,68	231,96
9	248,28	16,38	1 000	N/A	711 457	245,92	251,76

MUT: c.1545-168A>G

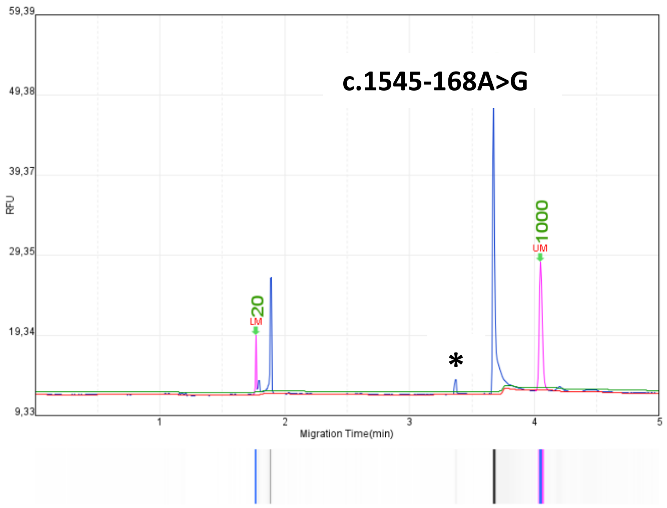

No	Time (sec.)	RFU	bp	Concn. (ng/μl)	PeakArea	PeakStart (sec.)	PeakEnd (sec.)
1	106,36	7,41	20	N/A	75 310	105,60	106,80
2	202,12	1,88	346	0,09	46 876	201,00	203,12
3	220,40	37,26	455	2,36	1 311 682	219,28	227,36
4	243,08	16,05	1 000	N/A	690 875	241,00	246,56

WT: c.1804-31C

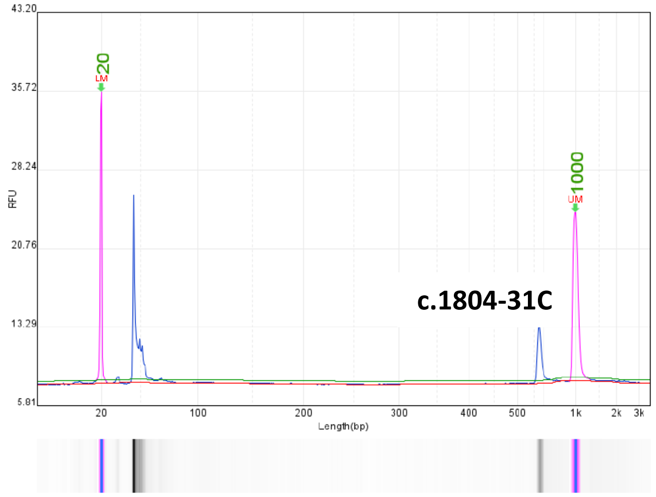

No	Time (sec.)	RFU	bp	Concn. (ng/μl)	PeakArea	PeakStart (sec.)	PeakEnd (sec.)
1	101.88	27.69	20	N/A	282,842	101.00	103.24
2	209.76	5.55	576	0.34	176,175	208.56	211.88
3	218.72	16.00	1,000	N/A	590,722	217.40	222.32

MUT: c.1804-31C>T

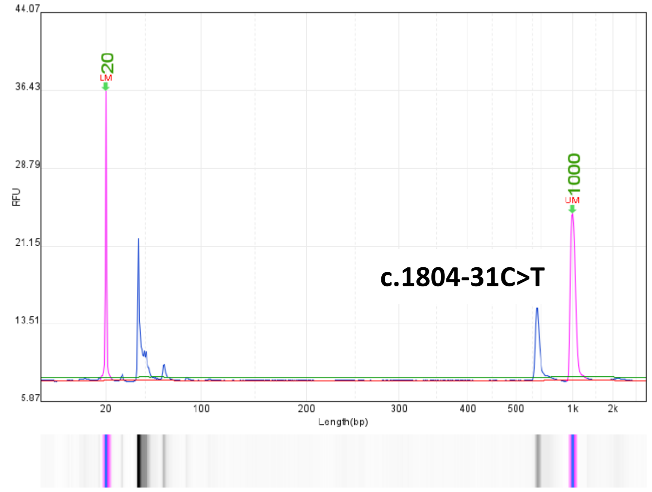

No	Time (sec.)	RFU	bp	Concn. (ng/μl)	PeakArea	PeakStart (sec.)	PeakEnd (sec.)
1	101.24	28.40	20	N/A	293,654	99.80	102.56
2	208.40	7.16	576	0.47	238,660	207.24	211.32
3	217.32	16.34	1,000	N/A	603,447	216.00	220.80

Figure S3. Capillary electrophoresis (Bio-Fragment Analyzer Qsep1) for PCR products for the wild type (WT) and mutant minigenes with variants c.942A>G, c.1545-168A>G, and c.1804-31C>T. The *SLC26A4* transcripts are visualized by the blue peaks. * – PCR products resulting from the activation of cryptic splicing sites in the genetic construct used. LM, UM – markers (20 bp and 1000 bp, respectively).

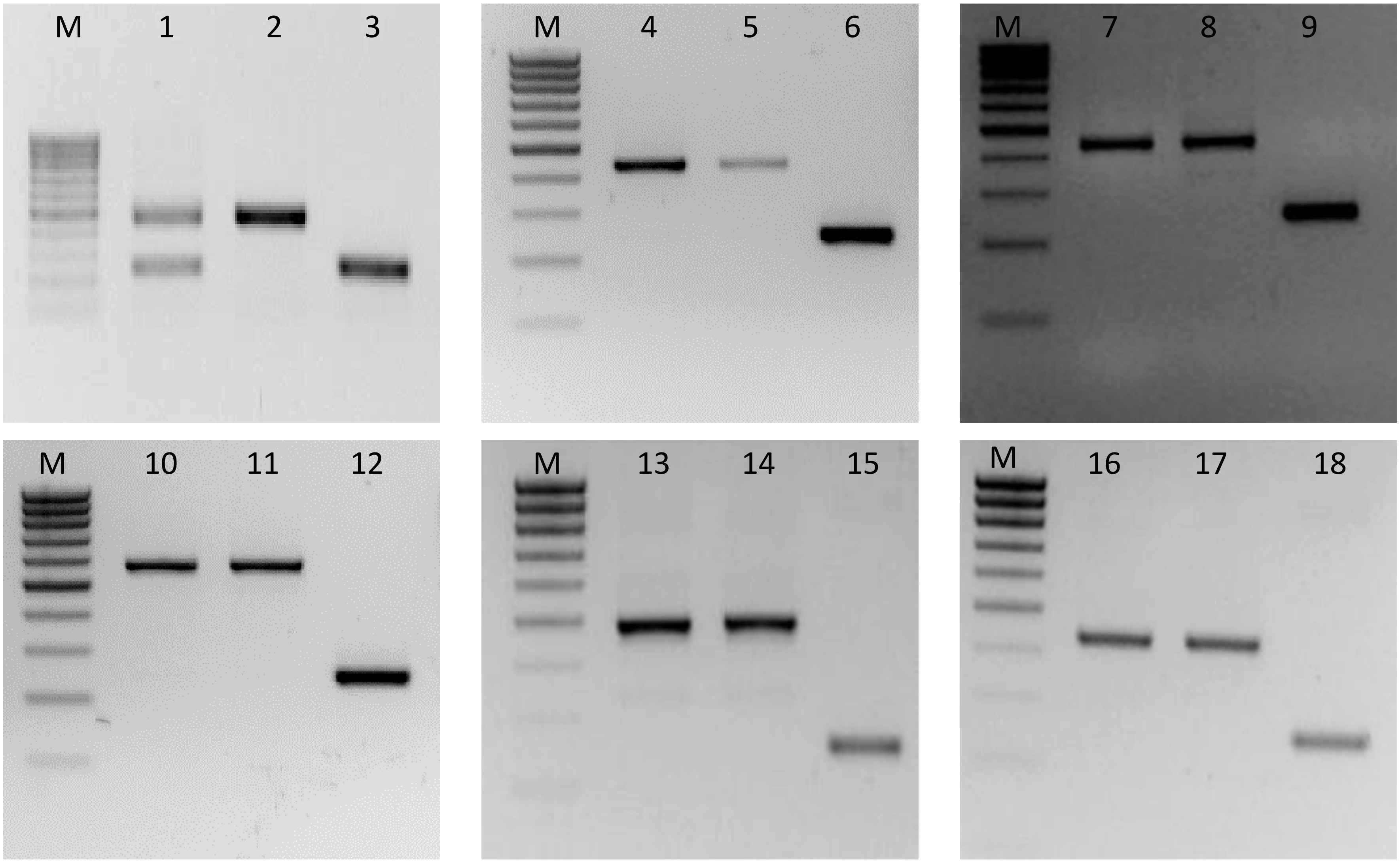

Figure S4. Experimental data obtained on the SW480 cell line. Agarose gel electrophoresis of PCR products for the wild type and mutant minigenes. pET01 – pET01 vector without insertion. M – 100 bp marker.

Line 1 – mt c.2034+1G>A (498 bp, 482 bp, 246 bp);
Line 2 – wt c.2034+1G (477 bp);
Line 3 – pET01 (246 bp);

Line 4 – wt c.1708-125T (435 bp);
Line 5 – mt c.1708-125T>C (435 bp);
Line 6 – pET01 (246 bp);

Line 7 – wt c.1708-18T (435 bp);
Line 8 – mt c.1708-18T>A (435 bp, 339 bp);
Line 9 – pET01 (246 bp);

Line 10 – wt c.1804-31C (573 bp);
Line 11 – mt c.1804-31C>T (573 bp);
Line 12 – pET01 (246 bp);

Line 13 – wt c.942A (482 bp);
Line 14 – mt c.942A>G (482 bp);
Line 15 – pET01 (246 bp);

Line 16 – wt c.1545-168A (423 bp);
Line 17 – mt c.1545-168A>G (423 bp);
Line 18 – pET01 (246 bp).
